# Blood Pressure Trajectories From Childhood to Youth and Arterial Stiffness in Adulthood: A 30-Year Longitudinal Follow-Up Study

**DOI:** 10.3389/fcvm.2022.894426

**Published:** 2022-06-29

**Authors:** Chao Chu, Yue-yuan Liao, Ming-jun He, Qiong Ma, Wen-ling Zheng, Yu Yan, Jia-wen Hu, Xian-jing Xu, Ya-ning Fan, Rui-hai Yang, Jian-jun Mu

**Affiliations:** ^1^Department of Cardiovascular Medicine, The First Affiliated Hospital of Xi’an Jiaotong University, Xi’an, China; ^2^Key Laboratory of Molecular Cardiology of Shaanxi Province, Xi’an, China; ^3^Department of Cardiovascular Medicine, Henan Province People’s Hospital, Zhengzhou, China; ^4^Institute of Cardiovascular Sciences, Hanzhong People’s Hospital, Hanzhong, China

**Keywords:** arterial stiffness, adolescent, risk factor, cohort study, blood pressure

## Abstract

**Background:**

This study aimed to identify the subgroups of individuals sharing similar blood pressure (BP) trajectories from childhood to youth and explore the associations of these trajectories with arterial stiffness in adulthood.

**Methods:**

A group-based trajectory model was used to identify BP trajectories among 2,082 individuals in the Hanzhong adolescent hypertension cohort by using BP values repeatedly measured at four visits from childhood (6–15 years) to youth (14–23 years). The brachial–ankle pulse wave velocity (baPWV) was examined 30 years after the baseline survey. Mixed linear regression models were used to examine the associations of these trajectories with adult baPWV.

**Results:**

Among the 2,082 individuals, three trajectory groups of systolic BP were identified as follows: the low-level group (*n* = 889), medium-level group (*n* = 1,021), and high-level group (*n* = 172). The baPWV in adulthood was higher in medium-level and high-level groups compared with the low-level group (1271.4 ± 224.7 cm/s, 1366.1 ± 249.8 cm/s vs. 1190.1 ± 220.3 cm/s, all *p* < 0.001). After adjustment for potential confounding factors, the association between baPWV and systolic BP trajectories was statistically significant (adjusted β = 49.4 cm/s; *p* < 0.001 for the medium-level group and β = 107.6 cm/s; *p* < 0.001 for the high-level group compared with the low-level group). Similar results were obtained for the association of baPWV with the trajectories of diastolic BP and mean arterial pressure (MAP), except for pulse pressure.

**Conclusion:**

Our investigation demonstrates different BP trajectories from childhood to youth and shows the trajectories of systolic BP, diastolic BP, and MAP are significant predictors of arterial stiffness in adulthood.

## Introduction

Increased arterial stiffness is increasingly recognized as a surrogate endpoint for cardiovascular disease (CVD) (1). As an indicator of arterial stiffness, brachial–ankle pulse wave velocity (baPWV), which is measured non-invasively and conveniently, has been widely used in clinical research. Longitudinal evidence showed that baPWV is an independent predictor of cardiovascular (CV) events and all-cause mortality (2). Given that CVD originates in early life, identifying early-life risk factors associated with long-term baPWV is of considerable importance in preventing CVD.

Blood pressure (BP) level is closely related to baPWV in the same period, whereas whether long-term baPWV can be independently predicted by BP in childhood remains controversial (3–5). It is noteworthy that, in previous studies, available adolescent BPs was primarily based on BP measurements at one-time point, which is easily affected by environmental and psychological factors. In addition, Li et al. (6) reported that the risk of stroke in individuals with similar baseline BP differs, further indicating that the use of BP at one-time point may cause misclassification of risk groups. Recent longitudinal studies opted to identify high-risk populations by using “BP trajectories” based on multiple BP measurements over years and assigned individuals into several subgroups that share similar patterns over time. Studies have shown that BP trajectories may be a stronger indicator of CVD compared with single BP assessment (6, 7). Tielemans et al. (8) reported that 10-year BP trajectories during middle age were strong predictors of CV mortality and all-cause mortality in a Minnesota study. Hao et al. (9) identified three BP trajectories from childhood to young adulthood in 683 individuals aged 5–16 years at the baseline and found that the trajectories are independently associated with both IMT and LVMI in adulthood. Zhang and colleagues found significant associations of childhood-to-adult BP trajectories with adult LVH and remodeling patterns in the Bogalusa heart study (10). More recently, we identified four distinct systolic BP trajectories from childhood to adulthood based on a longitudinal cohort study consisting of 4,623 individuals aged 6–15 years old at baseline and found a strong association between 30-year BP trajectories and the risk of subclinical renal damage in adulthood (11). However, the relationship between BP trajectories from childhood to youth and long-term arterial stiffness remains unclear.

In the present study, we aimed to identify the subgroups of individuals sharing similar BP trajectories from childhood to youth and explore the associations of the trajectories with baPWV in adulthood on the basis of longitudinal data from the Hanzhong adolescent hypertension cohort recruited in 1987.

## Materials and Methods

### Study Participants

The Hanzhong adolescent hypertension cohort is a prospective cohort study that recruited 4,623 school students aged 6–15 years old from 26 rural areas in three towns in Hanzhong City, Shaanxi, China, in 1987. Thereafter, this cohort was large-scale followed up in 1989, 1992, 1995, 2013, and 2017. A more thorough description of the study design and conduct has been previously published (11). Written informed consents were obtained from all participants at each visit, and for those under 18 years of age at baseline, consent from a parent/guardian was obtained. The study protocol was approved by the Academic Committee of the First Affiliated Hospital of Xi’an Jiaotong University (XJTU1AF2015LSL-047) and clinical registration number is NCT02734472.

In the latest follow up (in 2017), a total of 2,780 individuals were examined, and 1,832 individuals were absent (1,196 individuals were unable to participate or could not be contacted due to several reasons such as migration, non-local laborers, military services or mental illness, 554 people refused to attend, and 93 subjects died). During this period, baPWV measurement was performed in 2,461 individuals. After excluding 379 individuals with less than three BP measurements from childhood to youth (1987–1995), the remaining 2,082 participants were included in the analysis (Supplementary Figure 1).

### Physical Measurements

Demographic characteristics, personal/family medical history (such as hypertension, diabetes, hyperlipidemia and stroke), parents’ educational levels (at baseline), current medication use, family medical history, cigarette smoking, and drinking history were obtained through a self-administered questionnaire. Anthropometric indicators, including height, body weight and waist circumference were measured repeatedly during each visit, and the mean of each parameter was used for analysis. Height and weight were measured on individuals without shoes and wearing underwear. Height was measured to the nearest 0.2 cm and weight to the nearest 0.5 kg. BMI was calculated as kilograms per square meter (kg/m^2^). Cigarette smokers are defined by the World Health Organization (WHO) as individuals who smoke at least one cigarette daily for a continuous period of at least 6 months. Family history (FH) of hypertension at the baseline was defined as a minimum of one first- or second-degree relative suffering from hypertension.

### Biochemical Assays

Fasting venous blood samples were collected by experienced nurses in the morning after fasting at least 8 h and serum was isolated. Serum biochemical indicators, including fasting glucose, uric acid, triglyceride (TG), total cholesterol (TC), and high- and low-density lipoprotein cholesterols (HDL-C, LDL-C) were measured using a Hitachi 7060 automatic biochemical analyzer.

### Blood Pressure Measurements

Sitting blood pressure (BP) were measured at the baseline and during each follow-up period by using mercury (1987–2013) or electronic sphygmomanometer (2017, OMRON HBP-1100) by trained and certified staff members in accordance with the procedures recommended by the WHO or operating instructions in a quiet and comfortable environment (12). The appropriate cuff size was selected depending on their right upper arm circumference. Participants were required to avoid cigarette smoking, alcohol, coffee/tea, and strenuous exercise for at least 30 min before measuring. Three BP measurements were performed with an interval of 1 min, and the systolic BP (SBP) and diastolic BP (DBP) values were defined as the mean level of the second and third BP measurements. Mean arterial pressure (MAP) was defined as DBP + [(SBP-DBP)/3]. Pulse pressure (PP) was calculated as the difference between SBP and DBP.

### Evaluation of Arterial Stiffness

The baPWV was assessed using an automatic arteriosclerosis diagnostic device BP-203RPE III (Colin Co., Ltd., Komaki, Japan) in a quiet and comfortable environment. The operator encoded the following information of subjects in the user interface: name, gender, height, body mass, and date of birth. Measurements were performed on the subject in a supine position after a rest period of 5 min, with cuffs wrapped around both sides of the upper arm and ankle, electrocardiogram electrodes sandwiched both wrists, and a heart sound sensor placed on the fourth intercostal space left sterna border. PWV, BP, electrocardiogram, and heart sounds were simultaneously recorded by the instrument. The time interval between the upper arm and ankle (ΔT) was defined as the time interval between the wave front of the brachial waveform and that of the ankle waveform. The path length from the suprasternal notch to the brachium (Lb) or to the ankle at the same side (La) was automatically calculated according to the subject’s height using the following formulae (13): Lb (cm) = 0.2195 × height- 2.0734 and La (cm) = 0.8129 × height + 12.328. Finally, the bilateral baPWV was automatically calculated as follows: baPWV (cm/s) = (La–Lb)/ΔT. The average of both sides of baPWV was used for the analysis.

### Statistical Analysis

All analyses were conducted using Stata software version 12.1 (STATA Corp., TX) and SPSS16.0 (SPSS Inc., Chicago, Illinois, United States). BP trajectories were identified using group-based trajectory models within a Stata plugin program (Traj) (14). The model estimated the posterior probability of each individual in each trajectory subgroup and then assigned him/her into the subgroup with the highest posterior probability. In the analysis, BP trajectories were constructed with the values at four time points from 1987 to 1995 by using the individual’s age as a timescale. The model parameters were estimated by adopting a censored normal model appropriate for continuous variables, first without covariates, then height was included as a time-varying covariates. A model of one to five trajectories was successively considered. Polynomial terms of age (cubic, quadratic, or linear) were considered as the shape of each group, and the parsimonious model was determined by excluding polynomial terms that did not achieve statistical significance. Several indicators, including minimum Bayesian information criteria, average posterior probability (≥ 70%), and membership in each trajectory group (≥ 5%) were used to determine the best fitting model (15). Finally, the best-fit model comprised the three classes with up to cubic order terms (Supplementary Table 1).

The associations of BP trajectories with baPWV in adulthood were analyzed by mixed linear regression model with unstructured covariance. In the full adjusted model, BP trajectories were used as dummy variables, and the trajectory group with the lowest BP level was treated as reference. The number of visits was included as a random variable. Associations were adjusted for time-dependent variables [age, body mass index (BMI), SBP, DBP, MAP, and HR], biochemical indicators (fasting blood glucose, TC, TG, LDL-C, HDL-C, uric acid at the latest follow-up), and categorical variables (sex, smokers, FH of hypertension, and parental education levels). For observing whether interactions occur between gender and BP trajectory groups, gender × SBP trajectories and gender × DBP trajectories were incorporated into the corresponding overall association analysis. We also performed hierarchical analysis based on sex. In addition, to exclude the impact of medication use at the last follow up on the results, we performed sensitivity analysis after excluding individuals who were taking antihypertensives, hypoglycemics, or statins at the time (*N* = 86). A *p*-value < 0.05 was considered statistically significant in this study.

## Results

### Blood Pressure Trajectories From Childhood to Youth

**FIGURE 1:**
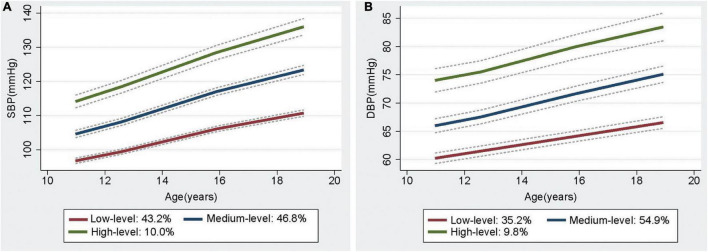
The trajectories of Systolic BP **(A)** and Diastolic BP **(B)** from childhood to youth, with 95% confidence intervals, in the Hanzhong adolescent hypertension cohort.

Three trajectories of BP from childhood to youth were identified, and each of the trajectories was labeled on the basis of their BP patterns over time, namely, low-, medium-, and high-level BP trajectories (Figure 1A and Supplementary Table 2). For the SBP trajectories, 43.2% (*n* = 889) of participants began with a low SBP, which increased from 1987 to 1995, but only up to around 110 mmHg (referred to as low-level group). Meanwhile, 46.8% (*n* = 1021) of the participants were measured with an SBP of 100–110 mmHg and manifested rapid BP increase over the 8-year period that remained < 125 mmHg (referred to as the medium-level group). Lastly, 10.0% (*n* = 172) commenced at high levels and experienced the highest increase in SBP throughout the observation period (referred to as the high-level group). Meanwhile, the three DBP trajectories were low-level (35.2%), medium-level (54.9%), and high-level (9.8%). The trajectories differed at the baseline and increased over time (Figure 1B and Supplementary Table 3). In addition, three trajectories of MAP and pulse pressure (PP) were also found in this population (Supplementary Figure 2).

To verify whether the initial BP trajectories of the participants were relatively stable over time, we further constructed a 30-year SBP trajectory based on the values at six visit time points from 1987 to 2017 (Supplementary Figure 3), and found that nearly 75% of individuals in the low-level and medium-level SBP trajectory from childhood to youth were still in the later low-stable and medium-stable group. Similarly, those in the initial high-level group were mostly in the medium-increasing group and high-stable group in adulthood (Supplementary Table 4), suggesting that individuals’ BP trajectories are relatively stable over time.

### General Characteristics of the Study Population

The characteristics of participants at baseline and the last follow-up based on different SBP trajectory groups are presented in Table 1. Individuals with the high-level SBP pattern were more likely to be men and smokers, and present high BP, BMI and HR at baseline as well as at the latest follow-up period. Meanwhile, the group of individuals had the highest concentrations of fasting glucose, serum uric acid, and the lowest level of HDL-C at the latest follow-up among all trajectories. Moreover, baPWV in adulthood was significantly different among the SBP trajectory groups, being lowest in the low-level group and highest in the high-level group (1190.1 ± 220.3 cm/s vs. 1271.4 ± 224.7 cm/s vs. 1366.1 ± 249.8 cm/s, all *p* < 0.001). Similar trends were found in the DBP and MAP trajectories, except for the PP trajectories (Supplementary Table 5).

**TABLE 1:** General Characteristics of the study participants at baseline and the last follow-up by systolic BP trajectory group.

Characteristics	Low-level group (*n* = 889)	Medium-level group (*n* = 1,021)	High-level group (*n* = 172)	*p*_–trend_
Male (%)	390 (43.9%)	591 (57.9%)	121 (70.3%)	<0.001
**Baseline**				
Age (years)	12.0 (9.0–13.0)	11.0 (9.0–13.0)	11.0 (8.0–13.0)	0.314
Systolic BP (mmHg)	97.8 ± 7.9	106.8 ± 9.0	115.3 ± 9.6	<0.001
Diastolic BP (mmHg)	61.8 ± 7.9	67.0 ± 8.8	71.8 ± 8.9	<0.001
BMI (kg/m^2^)	16.1 ± 2.1	16.5 ± 2.1	16.7 ± 2.2	<0.001
Heart rate (bpm)	78.0 ± 9.5	78.7 ± 9.7	81.4 ± 11.0	<0.001
**Father’s education level (%)**				
Primary or less	455 (51.1%)	508 (49.8%)	83 (48.3%)	0.711
Middle school	328 (36.9%)	380 (37.2%)	66 (38.4%)	0.934
High school or above	106 (12.0%)	133 (13.0%)	23 (13.4%)	0.729
**Mother’s education level (%)**				
Primary or less	688 (77.4%)	796 (78.0%)	134 (77.9%)	0.957
Middle school	169 (19.0%)	194 (19.0%)	33 (19.2%)	0.998
High school or above	32 (3.6%)	30 (2.9%)	5 (2.9%)	0.696
FH of hypertension (%)	73 (8.2%)	115 (11.3%)	18 (10.5%)	0.081
**Follow-up in 2017**				
Age (years)	42.0 (39.0–43.0)	41.0 (39.0–43.0)	41.0 (38.0–43.0)	0.314
Smokers, n (%)	320 (36.0%)	456 (44.7%)	88 (51.2%)	<0.001
FH of hypertension, n (%)	418 (47.0%)	560 (54.8%)	90 (52.3%)	0.280
Systolic BP (mmHg)	111.6 ± 15.5	125.0 ± 15.5	132.1 ± 16.4	<0.001
Diastolic BP (mmHg)	73.2 ± 11.1	78.3 ± 11.4	83.0 ± 11.5	<0.001
Heart rate (bpm)	73.6 ± 10.1	73.6 ± 10.0	75.4 ± 11.1	0.094
BMI (kg/m^2^)	23.8 ± 3.2	24.2 ± 3.2	24.9 ± 3.2	<0.001
Triglycerides (mmol/L)	1.31 (0.94–1.89)	1.32 (0.94–1.92)	1.44 (1.01–2.02)	0.074
TC (mmol/L)	4.57 ± 0.81	4.51 ± 0.80	4.61 ± 0.76	0.135
LDL-C (mmol/L)	2.54 ± 0.67	2.49 ± 0.64	2.61 ± 0.59	0.056
HDL-C (mmol/L)	1.20 ± 0.27	1.17 ± 0.27	1.14 ± 0.23	0.012
Fasting glucose (mmol/L)	4.54 (4.27–4.85)	4.59 (4.27–4.91)	4.72 (4.35–5.09)	0.001
Serum uric acid (mmol/L)	275.9 ± 74.6	287.2 ± 78.8	296.9 ± 84.0	<0.001

*Continuous variables were expressed as mean ± SD or Median (P25,P75), and groups differences were compared using the t-test or Mann–Whitney U test according to the normality of distribution. Categorical variables were expressed as percentages, and group differences were tested with χ^2^-test. BP, blood pressure; BMI, body mass index; FH of hypertension, family history of hypertension; TC, total cholesterol; LDL-C, low density lipoprotein-cholesterol; HDL-C, high density lipoprotein cholesterol.*

### Association of Brachial–Ankle Pulse Wave Velocity With Blood Pressure Trajectories

Table 2 shows the results of mixed linear regression analysis of BP (SBP and DBP) trajectories from childhood to youth on baPWV in adulthood. Higher BP trajectory levels were associated with higher baPWV (*p*_trend_ < 0.001). After adjustment for potential confounding factors in early life, the association between baPWV and the SBP trajectories remained statistically significant (an average increase of 49.4 cm/s and 107.6 cm/s for baPWV were respectively, observed in the medium-level group and the high-level group compared with the low-level group, all *p* < 0.001). For DBP trajectories, the trajectory level was a significant predictor of arterial stiffness in adulthood (adjusted β = 26.8 cm/s for the medium-level group and β = 103.8 cm/s for the high-level group compared with the low-level group, all *p* < 0.001). Furthermore, no interaction effect was found between sex and SBP or DBP trajectories in the overall association analysis (all *p* > 0.05).

**TABLE 2:** Associations of baPWV in adulthood with BP trajectories from childhood to youth.

Trajectories	N	Model1		Model2		Model3	
		β (95%CI)	*P*-value	β (95%CI)	*P*-value	β (95%CI)	*P*-value
**SBP trajectory groups**							
Low-level	889	Reference		Reference		Reference	
Medium-level	1,021	81.3 (71.2–91.4)	<0.001	65.5 (55.8–75.2)	<0.001	49.4 (37.9–61.1)	<0.001
High-level	172	176.0 (157.7–194.4)	<0.001	147.7 (130.0–165.3)	<0.001	107.6 (85.9–129.3)	<0.001
**DBP trajectory groups**							
Low-level	692	Reference		Reference		Reference	
Medium-level	1,245	47.2 (36.6–57.8)	<0.001	43.4 (33.3–53.4)	<0.001	26.8 (15.1–38.6)	<0.001
High-level	145	138.3 (117.9–158.8)	<0.001	140.0 (120.6–159.3)	<0.001	103.8 (80.9–126.7)	<0.001

*baPWV, brachial–ankle pulse wave velocity; SBP, systolic blood pressure; DBP, diastolic blood pressure. CI, confidence interval. Dependent variables: baPWV. Model1: Unadjusted; Model2: Adjusted for age, sex (male = 1, female = 0); Model3: adjustments as in model2 plus time-dependent variables (body mass index, SBP, DBP, MAP and heart rate), childhood factors including father’s education level and mother’s education level (College or university = 0, High school = 1, Middle school = 2 and Primary or less = 3) and adulthood factors including family history of hypertension (Yes = 1, No = 0), smokers (Yes = 1, No = 0), TC, TG, LDL-C, HDL-C, uric acid, and fasting blood glucose.*

We also analyzed the associations between adulthood baPWV and the trajectories of MAP and PP, and found that after adjusting for confounding factors, the MAP trajectory levels were strong predictors of long-term arterial stiffness (all *p* < 0.001). Additionally, a significant positive association was observed between baPWV with PP trajectory levels when adjusted for gender and age. However, this association completely disappeared after further adjusting for the time-variant variable SBP (Supplementary Table 6).

### Sensitivity Analysis

**FIGURE 2:**
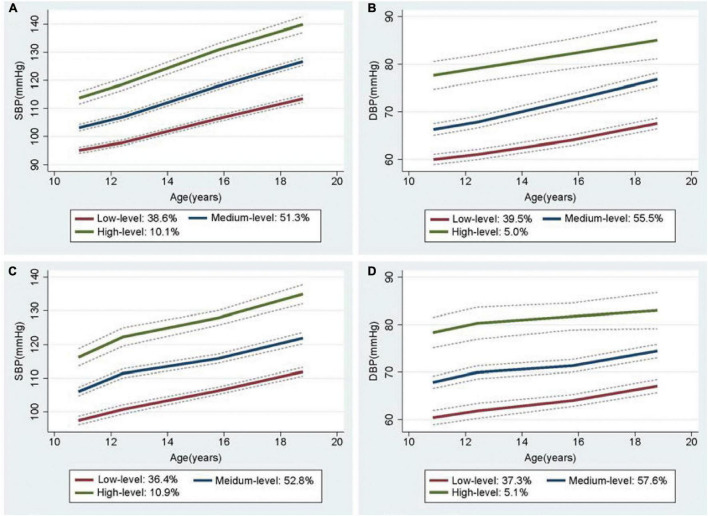
Trajectory groups in systolic blood pressure (SBP) and Diastolic blood pressure (DBP) for male from adulthood to youth in the Hanzhong adolescent hypertension cohort. **(A)** SBP over time, **(B)** DBP over time, **(C)** SBP over time adjusted for height as a time-varying covariate, **(D)** DBP over time adjusted for height as a time-varying covariate.

**FIGURE 3:**
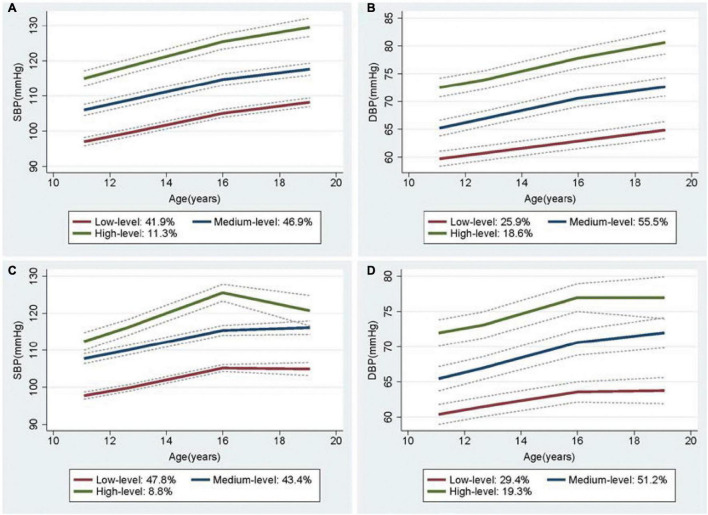
Trajectory groups in systolic blood pressure (SBP) and Diastolic blood pressure (DBP) for female from adulthood to youth in the Hanzhong adolescent hypertension cohort. **(A)** SBP over time, **(B)** DBP over time, **(C)** SBP over time adjusted for height as a time-varying covariate, **(D)** DBP over time adjusted for height as a time-varying covariate.

In a sex-stratified analysis, three trajectories of SBP and DBP with cubic terms were also identified in both genders (Figures 2A,B, 3A,B). Although the BP levels in the different trajectories showed an increasing trend over time in both sexes, the men tended to show lower initial BP levels and faster BP growth rates than women, and men showed higher BP levels after 8 years (Supplementary Figure 4). In addition, to explore the effect of height on BP trajectories from childhood to youth in different genders, we further constructed the BP trajectories using height from 1987 to 1995 as a time-varying covariate, and the results showed only a slight change in the proportion of individuals in each trajectory group, except for a nearly 6% increase in the low-level SBP group in female (Figures 2C,D, 3C,D). Moreover, height from childhood to youth was positively associated with all BP trajectories in both genders (Supplementary Table 7). Further association analysis showed that, consistent with the overall analysis, the child-to-youth BP trajectories were significantly associated with long-term baPWV in both sexes except for PP trajectories (Supplementary Table 8).

Arterial stiffness can be improved by several medications, including antihypertensive and hypoglycemic drugs or statins (16). We performed further analysis after excluding individuals who are currently consuming antihypertensives, hypoglycemic drugs, or lipid-lowering drugs (*n* = 86). No significant change in the values and characteristics of BP trajectories were noted (Supplementary Figure 5). Meanwhile, as shown in Table 3, the associations of baPWV with other SBP and DBP trajectory groups were weakened but still remained statistically significant in the subgroup individuals (adjusted β = 52.4 cm/s, *p* < 0.001 and β = 24.6 cm/s, *p* < 0.001 for the medium-level group and β = 81.8 cm/s, *p* < 0.001 and β = 98.7 cm/s, *p* < 0.001 for the high-level group compared with the low-level group for SBP and DBP trajectory groups, respectively). In addition, the MAP trajectory groups, but not the PP trajectory groups, were also independently associated with long-term arterial stiffness.

**TABLE 3:** Sensitivity analysis of baPWV affected by different BP trajectories from childhood to youth when individuals currently taking antihypertensive, hypoglycemic or statins medicine were excluded (*N* = 86).

Trajectories	N	Model1		Model2		Model3	
		β (95%CI)	*p*-value	β (95%CI)	*p*-value	β (95%CI)	*p*-value
**SBP trajectory groups**							
Low-level	845	Reference		Reference		Reference	
Medium-level	971	82.9 (73.1–92.7)	<0.001	66.1 (56.7–75.4)	<0.001	52.4 (41.3–63.6)	<0.001
High-level	180	141.5 (124.4–158.7)	<0.001	116.7 (100.4–133.0)	<0.001	81.8 (61.6–102.0)	<0.001
**DBP trajectory groups**							
Low-level	610	Reference		Reference		Reference	
Medium-level	1,232	41.5 (31.0–51.9)	<0.001	37.9 (28.1–47.7)	<0.001	24.6 (13.1–36.0)	<0.001
High-level	154	127.7 (108.7–146.8)	<0.001	123.0 (112.1–147.8)	<0.001	98.7 (77.2–120.2)	<0.001
**MAP trajectory groups**							
Low-level	832	Reference		Reference		Reference	
Medium-level	1,022	77.0 (67.2–86.7)	<0.001	66.1 (57.0–75.3)	<0.001	57.5 (46.5–68.6)	<0.001
High-level	142	152.7 (132.8–172.6)	<0.001	155.0 (136.3–173.7)	<0.001	139.6 (117.4–161.8)	<0.001
**PPtrajectory groups**							
Low-level	1,456	Reference		Reference		Reference	
Medium-level	463	55.3 (43.9–66.7)	< 0.001	32.6 (21.7–43.4)	<0.001	12.3 (−0.1–24.8)	0.052
High-level	77	82.2 (58.5–105.9)	< 0.001	30.4 (7.8–52.9)	0.008	−0.6 (−24.9–23.7)	0.963

*baPWV, brachial–ankle pulse wave velocity; SBP, systolic blood pressure; DBP, diastolic blood pressure. MAP, mean arterial pressure; PP, pulse pressure. Dependent variables: baPWV. Model1: Unadjusted; Model2: Adjusted for age, sex (male = 1, female = 0); Model3: adjustments as in model2 plus time-dependent variables (body mass index, SBP, DBP, MAP and heart rate), childhood factors including father’s education level and mother’s education level (College or university = 0, High school = 1, Middle school = 2 and Primary or less = 3) and adulthood factors including family history of hypertension (Yes = 1, No = 0), smokers (Yes = 1, No = 0), TC, TG, LDL-C, HDL-C, uric acid, and fasting blood glucose.*

## Discussion

In a perspective cohort of 2,082 Chinese children and adolescents, we identified three trajectories for SBP and DBP from childhood to youth using BP measurements at four time points over 8-year period. In addition, we conducted a 30-year follow-up study and found that high BP trajectories from childhood to youth were associated with high baPWV in adulthood. Identifying BP trajectories from childhood to youth may provide an effective approach to distinguish populations with increased risk for developing CVD.

Recently, increasing attention has been focused on the BP trajectory because of its potential role in cardio- and cerebrovascular diseases and end points (6, 17). At present, BP trajectories from childhood to young adulthood have been investigated in several studies (9, 18, 19). In the Georgia Stress and Heart study, three trajectories of BP (SBP, DBP, and mid-BP) during childhood were identified in 626 individuals, who presented a minimum of three BP measurements before 18 years of age (9). Theodore et al. constructed four SBP trajectories from childhood to early midlife in a New Zealand birth cohort consisting of 975 individuals born from 1972 to 1973 by using six BP measurements from 7 years to 38 years (18). Moreover, Wang et al. (19) reported that different ambulatory BP trajectories were identified among 663 Afro- and European–American males and females aged 7 years to 30 years on the basis of a 12-time data collection (five times on average) over a 15-year longitudinal study. As indicated by the above-mentioned studies, the values and characteristics of the BP trajectory varied with changes in several factors, including age, gender, race, and time points of BP measurement. We explored the BP trajectories from childhood to youth in a cohort of children and adolescents in rural China for the first time. Three trajectories of BP (SBP, DBP, MAP, and PP) were identified using three or four BP measurements from an average of 11 years to 19 years of age. In our study, the BP of each trajectory group rapidly increased with age and height in this age group, and gender differences existed in the BP trajectories. Overall, the men showed lower initial BP levels, faster BP growth rates, and higher terminal BPs than women within 11–19 years of age. These findings were consistent with the previously mentioned results and in line with the development law of BP in puberty (20). We also observed that the shape and slope of the curves were not identical between different trajectories. Meanwhile, individuals with high-level SBP pattern were more likely to be men and present high BMIs and HRs. These results further illustrated the heterogeneity in BP transitions over time in children and adolescents.

To date, the relationship between BP trajectory in early life and long-term arterial stiffness remains unclear. To our knowledge, this study is the first to illustrate the issue. Our results showed that baPWV in adulthood was higher in medium-level and high-level groups compared with the low-level group. More importantly, after adjustment for age, sex, SBP, DBP, and current medication use, high SBP trajectories from childhood to youth were identified as significant predictors of increased adult baPWV. The results obtained seem to be best explained by the relationship of arterial stiffness with BP load and cumulative burden over time. Aatola et al. (4) demonstrated that the risk of high adult PWV increased by 3.1 times in individuals with persistent elevated BP in both childhood and adulthood than those with sustained normal BP in the CV risk in a study involving young Finns. Meng et al. (21) reported that individuals with sustained hypertension presented the risk of developing arterial stiffness compared with those with normal BP and non-sustained hypertensive individuals in a 2-year longitudinal study consisting of 236 hypertensive and non-hypertensive children. Moreover, Tedla and his colleagues conducted a 9.5-year follow-up study in the multiethnic study of atherosclerosis and found that extended duration of effective BP control was associated with decreased progression in arterial stiffness (22). Several longitudinal studies have explored the temporal relationship between BP and arterial stiffness, and the results suggested that higher artery stiffness was associated with incident hypertension, while elevated BP can further resulted in increased arterial stiffness (23–25). It can be seen that BP and arterial stiffness were both causal and mutually reinforcing. In terms of mechanism, arterial walls stiffen with age, then increased arterial stiffness acts on the BP and leads to further BP elevation. Meanwhile, long-term exposure to elevated BP mainly causes increased abnormal collagen content and decreased normal elastin in the aortic wall, leading to vascular stiffness (26). These results emphasize that the regular screening and long-term appropriate treatment of elevated BP in childhood is of considerable importance in the primary prevention of CVD.

In view of the significant impact of higher BP trajectories on long-term arteriosclerosis and the developmental heterogeneity in BP trajectories over time, it is particularly important to explore the various risk factors that differentiate between normal and pathological BP trajectories early in life. Height is known to be a main determinant of BP in children and adolescents, and is commonly used in the assessment of childhood BP status and the standardization of PWV (27, 28). To date, relatively few studies have examined the relationship between body height and BP trajectories in early life. Using data from the Bt20 cohort study, Kagura et al. show that height between ages 5 and 18 years was positively associated with all BP trajectories in both genders, except for the girls’ upper SBP and DBP trajectories (29). Gao et al. (30) reported that the upward shift of BP trajectories among Chinese youths was largely attributed to increasing height based on the longitudinal follow-up data of 3,785 children in the China Health and Nutrition Survey 1991–2015. Similar to the above findings, we adjusted for the individual’s height when analyzing latent BP trajectories in early life and found that BP tends to increase with age and height, and height from childhood to youth was positively associated with all BP trajectories in both genders. Mechanistically, the involvement of childhood height in BP regulation may be mainly through altering vascular structure and function (31). In addition to height, we also found that individuals with higher BMIs or smoking behavior were more likely to be in the high-level BP trajectory. A cohort study of 975 New Zealanders showed that increasing BMI and cigarette smoking both predicted an upward shift in BP trajectory levels from childhood to early-midlife (18). Interestingly, evidence from the Childhood Determinants of Adult Health Study indicated that decreased BMI and increased vegetable consumption between childhood to adulthood could promote the transition from childhood hypertension to normal adult BP (32). These findings further underscore the important value of lifestyle improvements for cardiovascular disease risk reduction in the general population (33). In addition, several longitudinal studies have reported that the parameters affecting the shift between normal and pathological BP trajectories and the occurrence of adulthood hypertension also included some established risk factors (e.g., male sex, ethnicity, family history of hypertension, parity, low birth weight, rapid growth in infancy, etc.) and other modifiable risk factors (e.g., salt intake, education level and physical activity, etc.) (7, 18, 19, 28, 31, 34–37). More perspective studies are needed to confirm these findings. It is worth noting that early interventions targeting these modifiable factors might help to prevent the development of long-term hypertension.

Several limitations exist in the current study. First, since this study were followed up for 30 years, combined with the study population were in young-adult stage, with great daily liquidity, resulting in high dropout rate during the last two visits. Second, considering the study conditions, biochemical indicators (fasting glucose, blood lipids, and uric acid) from childhood to youth were unavailable for discounting the potential confounding effects of metabolic factors. Finally, the study population comprised a Chinese Han population from rural areas of China. The baseline age was 6–15 years. BP trajectories are affected by factors, such as race, region, and age; therefore, the extrapolation of the results to other homogenous populations should be performed with caution.

In summary, different BP trajectories exist from childhood to youth, and the trajectories of SBP, DBP, and MAP are significant predictors of arterial stiffness in adulthood. Monitoring BP trajectories from childhood to youth may provide an effective approach for distinguishing populations with increased risk for developing CVD. Further study needs to optimize the time taken to identify common BP trajectories and determine high-risk individuals for carrying out targeted prevention as early as possible.

## Data Availability Statement

The original contributions presented in the study are included in the article/Supplementary Material, further inquiries can be directed to the corresponding author/s.

## Ethics Statement

The studies involving human participants were reviewed and approved by the Academic Committee of the First Affiliated Hospital of Xi’an Jiaotong University. Written informed consents were obtained from all participants at each visit, and for those under 18 years of age at baseline, consent from a parent/guardian was obtained.

## Author Contributions

CC and J-JM were responsible for designing the study and writing of the manuscript. CC, Y-YL, M-JH, QM, W-LZ, YY, J-WH, X-JX, and Y-NF performed the follow-up of this cohort and collected the data. Y-YL and M-JH analyzed the data and revised the manuscript. R-HY and J-JM were responsible for research organization and coordination. All authors read and accepted the final manuscript.

## Conflict of Interest

The authors declare that the research was conducted in the absence of any commercial or financial relationships that could be construed as a potential conflict of interest.

## Publisher’s Note

All claims expressed in this article are solely those of the authors and do not necessarily represent those of their affiliated organizations, or those of the publisher, the editors and the reviewers. Any product that may be evaluated in this article, or claim that may be made by its manufacturer, is not guaranteed or endorsed by the publisher.
