# Supplementary material for: Blood Pressure Trajectories From Childhood to Youth and Arterial Stiffness in Adulthood: A 30-Year Longitudinal Follow-Up Study
Source: Front Cardiovasc Med. 2022 Jun 29;9:894426. doi: 10.3389/fcvm.2022.894426 (PMC9278647; doi:10.3389/fcvm.2022.894426)
Supplement: Supplementary file 1 [file Data_Sheet_1.PDF]

Supplementary Material

1 Supplementary Figures

1.1 Figure S1

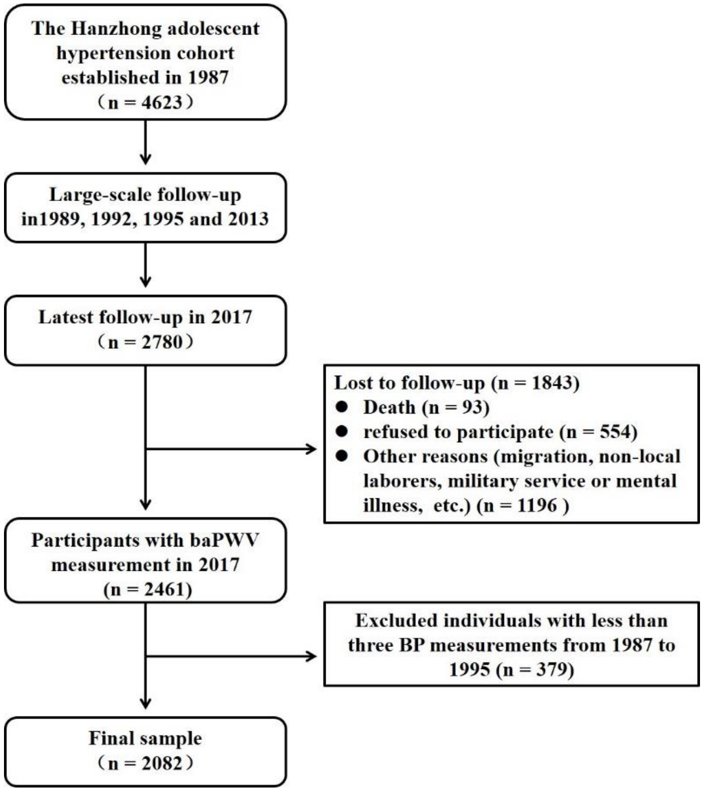

Figure S1 Flow diagram showing the selection of the study population.

1.2 Figure S2

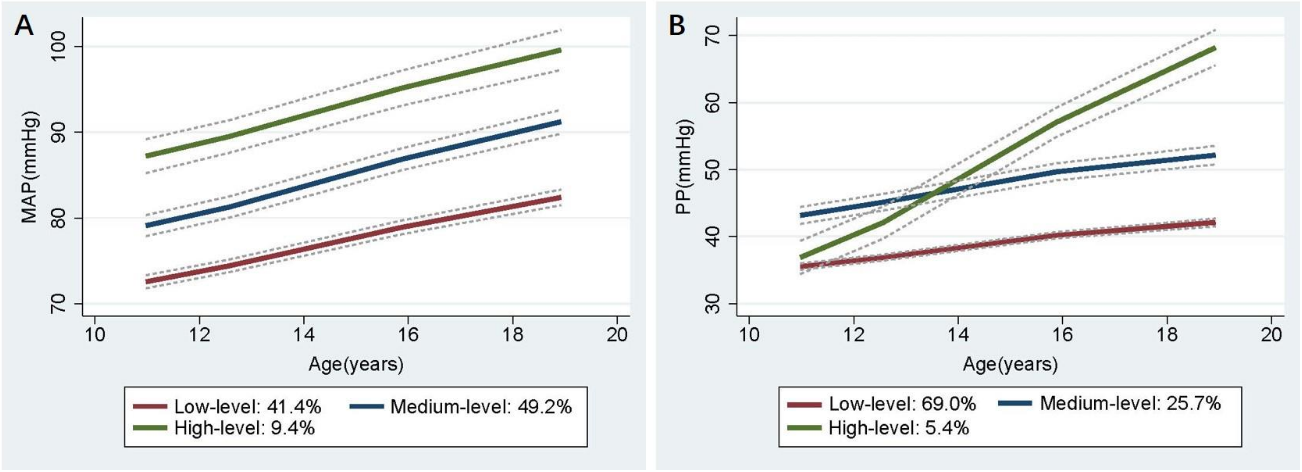

Figure S2 The trajectories of Mean arterial pressure (A) and Pulse pressure (B) from childhood to youth in the Hanzhong adolescent hypertension cohort

1.3 Figure S3

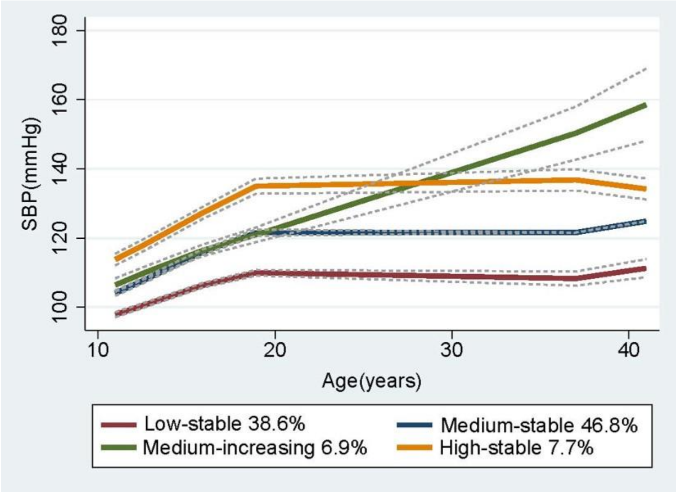

Figure S3 The trajectories of SBP from childhood to adulthood

in the Hanzhong adolescent hypertension cohort

1.4 Figure S4

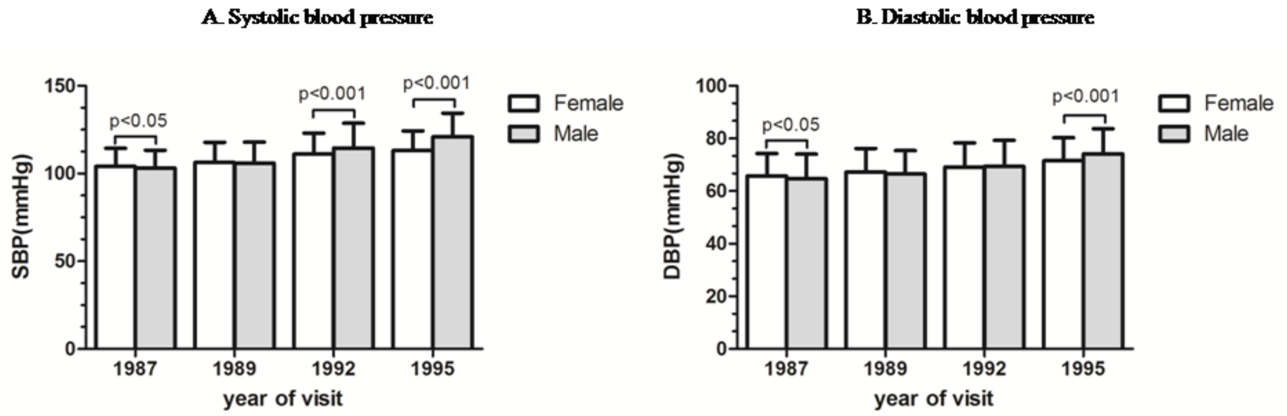

Figure S4 Comparison of SBP (A) and DBP (B) between sex during each visit between 1987 and 1995

1.5 Figure S5

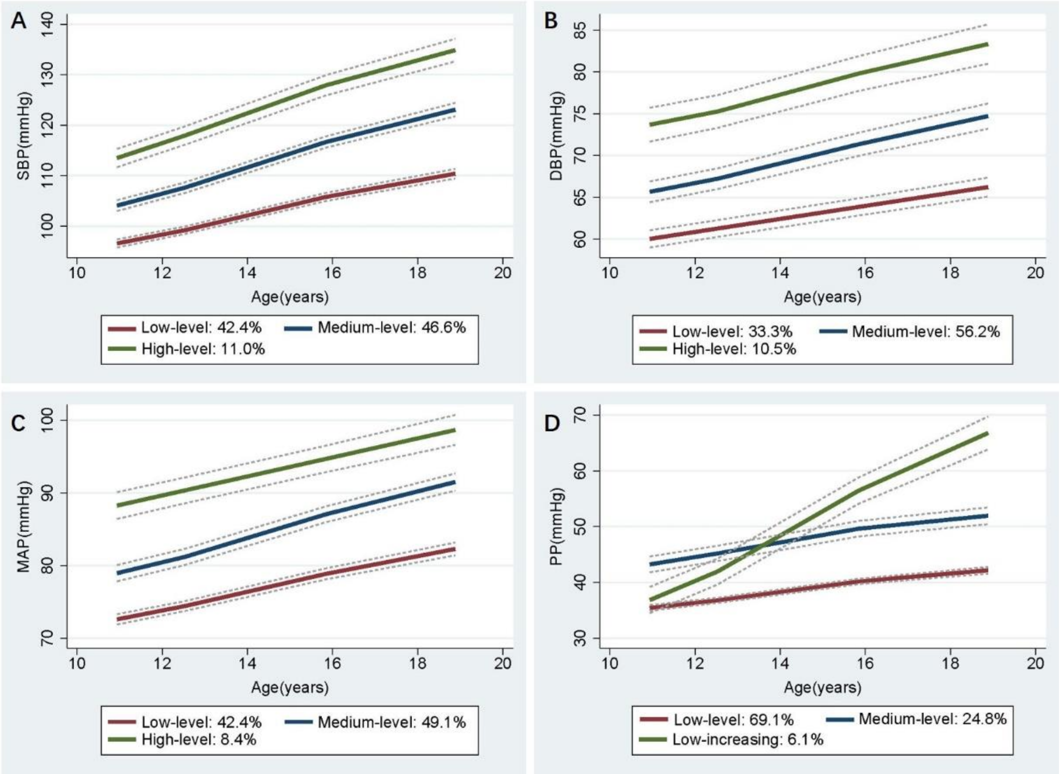

Figure S5 Trajectories of Systolic blood pressure (A), Diastolic blood pressure (B), Mean arterial pressure (C) and pulse pressure (D) in the population when individuals currently taking antihypertensive, hypoglycemic or statins medicine were excluded (N=86)

2 Supplementary Tables

2.1 **Table S1** Summary of group-based trajectory models (GBTM) fits

class No.	Polynomial degree	SBP trajectory-related parameters			DBP trajectory-related parameters		
		-BIC	Participants per class (%)	Posterior probabilities (%)	-BIC	Participants per class (%)	Posterior probabilities (%)
1	Linear	29709.49	100.0	NA	27840.55	100	NA
	Quadratic	29710.73	100.0	NA	27844.03	100	NA
	Cubic	29627.45	100.0	NA	27834.44	100	NA
2	Linear	29242.52	61.3/38.7	90.32/85.51	27565.28	59.3/40.7	86.53/82.61
	Quadratic	29248.57	61.3/38.7	90.19/85.67	27570.68	59.3/40.7	86.56/82.64
	Cubic	29128.06	60.7/39.3	90.37/86.66	27555.18	59.3/40.7	86.82/82.63
**3**	Linear	29183.63	44.8/46.6/8.6	84.78/79.10/79.93	27537.28	37.37/54.22/8.4	78.59/77.58/77.32
	Quadratic	29194.01	44.9/46.6/8.5	84.74/79.22/79.51	27546.23	37.7/54.1/8.2	78.72/77.85/76.85
	**Cubic**	**29066.53**	**43.2/46.8/10.0**	**85.23/79.29/80.20**	**27526.83**	**35.2/54.9/9.8**	**78.25/77.17/77.18**
4	Linear	29181.61	22.1/44.1/30.3/3.5	71.96/68.08/75.53/74.50	27540.62	31.0/50.1/9.3/9.6	72.81/71.54/53.15/74.54
	Quadratic	29195.54	43.3/46.1/8.2/2.4	84.14/77.33/72.36/65.54	27551.34	28.5/49.1/12.2/10.2	70.67/70.79/55.73/75.52

	Cubic	29052.57	40.8/33.2/18.4/7.6	81.58/67.54/64.71/80.42	27537.23	28.3/50.2/10.8/10.7	72.67/72.09/59.47/73.73
5	Linear	29183.54	29.3/41.7/24.4/2.7/2.0	75.67/66.41/71.70/66.68/77.99	27546.33	28.0/46.7/12.9/10.6/1.9	70.13/67.10/52.30/64.18/77.24
	Quadratic	29174.27	37.7/20.2/31.8/7.8/2.6	78.76/61.11/64.17/70.50/64.23	27554.89	18.2/40.3/21.6/17.5/2.4	65.74/61.44/65.53/56.46/74.66
	Cubic	29054.30	39.0/19.2/2.6/32.5/6.8	81.15/64.90/69.11/65.77/71.51	27544.41	19.1/43.6/20.4/14.6/2.3	67.70/64.48/65.74/57.70/73.12

The best fitting model is highlighted in bold characters. BIC: Bayesian information criterion, NA: not applicable.

2.2 **Table S2** Average SBP and DBP (mmHg) at each visit in SBP trajectory groups

Visit points	SBP (mmHg)			DBP (mmHg)		
	Low-level	Medium-level	High-level	Low-level	Medium-level	High-level
baseline	97.8±7.9	106.8±9.0	115.3±9.6	61.8±7.9	67.0±8.8	71.8±8.9
2-year follow-up	99.5±9.4	109.4±9.7	120.8±11.9	62.8±7.8	69.1±8.3	74.6±8.8
5-year follow-up	105.1±10.1	116.7±11.4	130.1±11.6	65.7±8.3	71.1±9.4	76.2±9.8
8-year follow-up	107.9±9.2	122.1±9.6	136.0±10.7	68.7±7.6	75.2±9.0	80.2±9.6

Abbreviation: SBP, systolic blood pressure; DBP, diastolic blood pressure.

2.3 **Table S3** Average SBP and DBP (mmHg) at each visit in DBP trajectory groups

Visit points	SBP (mmHg)			DBP (mmHg)		
	Low-level	Medium-level	High-level	Low-level	Medium-level	High-level
baseline	98.9±9.2	105.2±9.6	112.8±9.4	58.9±7.4	67.2±7.5	77.2±7.1
2-year follow-up	100.7±10.5	107.9±11.1	116.3±11.4	60.7±7.1	68.8±7.5	78.7±8.2
5-year follow-up	106.9±12.3	114.8±12.5	124.4±11.1	62.3±7.0	71.7±8.2	81.0±8.9
8-year follow-up	110.6±11.6	119.4±12.0	129.2±12.1	66.2±7.3	75.2±7.8	84.7±7.8

Abbreviation: SBP, systolic blood pressure; DBP, diastolic blood pressure.

2.4 **Table S4** Number of subjects distributed in 8-year or 30-year SBP trajectories

SBP trajectories from childhood to youth (8 years)	SBP trajectories from childhood to adulthood (30 years)			
	Low-stable	Medium-stable	Medium-increasing	High-stable
Low-level	661	191	37	-
Medium-level	140	756	82	43
High-level	-	59	12	101

Abbreviation: SBP, systolic blood pressure.

2.5 **Table S5** Associations of baPWV in adulthood with MAP and PP trajectories from childhood to youth

Trajectories	baPWV in adulthood (cm/s)		
	All subjects	Male	Female
SBP trajectory groups			
Low-level	1190.1±220.3	1268.6±220.4	1122.9±183.9
Medium-level	1271.4±224.7*	1321.6±217.5*	1197.0±233.4*
High-level	1366.1±249.8*#	1397.9±223.2*$	1272.7±231.8*$
DBP trajectory groups			
Low-level	1206.7±212.9	1268.1±201.1	1120.5±183.3
Medium-level	1253.9±231.9*	1324.3±227.5*	1175.0±221.3&
High-level	1344.5±268.9*#	1485.8±269.9*#	1241.4±237.3*#
MAP trajectory groups			
Low-level	1193.1±203.0	1273.2±213.2	1129.9±186.9
Medium-level	1269.4±236.6*	1323.1±216.8*	1184.8±224.9*
High-level	1358.4±269.5*#	1522.5±223.2*#	1261.2±253.8*$
PP trajectory groups			
Low-level	1228.0±232.7	1293.0±233.3	1165.9±218.1
Medium-level	1283.5±218.2*	1320.8±208.1&	1201.4±221.0
High-level	1319.4±236.3*	1354.9±226.6&	1200.5±218.4

SBP, systolic blood pressure; DBP, diastolic blood pressure; MAP, mean arterial pressure; PP, pulse pressure. *$P < 0.001$, and &$P < 0.05$ *vs*. Low-level group; #$P < 0.001$, and $\$P < 0.05$ *vs*. Medium-level group;

2.6 **Table S6** Associations of baPWV in adulthood with MAP and PP trajectories from childhood to youth

Trajectories	N	Model1		Model2		Model3	
		β(95%CI)	p-value	β(95%CI)	p-value	β(95%CI)	p-value
MAP trajectory groups							
Low-level	856	Reference		Reference		Reference	
Medium-level	1074	76.3(66.2-86.5)	<0.001	67.8(58.2-77.4)	<0.001	55.5(44.0-67.0)	<0.001
High-level	152	165.3(145.8-184.8)	<0.001	158.8(140.4-177.3)	<0.001	139.3(117.1-161.4)	<0.001
PP trajectory groups							
Low-level	1515	Reference		Reference		Reference	
Medium-level	485	55.5(43.7-67.2)	<0.001	31.8(20.6-43.1)	<0.001	4.0(-8.1-16.1)	0.519
High-level	82	91.3(65.8-116.8)	<0.001	40.9(16.4-62.3)	0.001	6.3(-19.4-32.0)	0.630

Abbreviation: baPWV, brachial–ankle pulse wave velocity; MAP, mean arterial pressure; PP, pulse pressure. CI, confidence interval.

Dependent variables: baPWV. Model1: Unadjusted; Model2: Adjusted for age, sex (male=1, female=0); Model3: adjustments as in model2 plus time-dependent variables (body mass index, SBP, DBP, MAP and heart rate), childhood factors including father's education level and mother's education level (College or university=0, High school=1, Middle school=2 and Primary or less=3) and adulthood factors including family history of hypertension(Yes=1, No=0) , smokers(Yes=1, No=0), TC, TG, LDL-C, HDL-C, uric acid, and fasting blood glucose.

2.7 **Table S7** Association of age terms and height between average age of 11 and 19 years with child-to-youth SBP trajectories

Group	Parameter	Estimate	SE	T value	P value
Low-level trajectory	Intercept	114.77911	7.08207	16.207	0.0000
	age	-12.46207	1.51996	-8.199	0.0000
	age2	0.87104	0.10819	8.051	0.0000
	age3	-0.01793	0.00247	-7.272	0.0000
	height	0.33068	0.02539	13.024	0.0000
Medium-level trajectory	Intercept	107.22189	5.65892	18.947	0.0000
	age	-12.55749	1.25724	-9.988	0.0000
	age2	0.82565	0.08990	9.184	0.0000
	age3	-0.01666	0.00206	-8.095	0.0000
	height	0.36394	0.02635	13.810	0.0000
High-level trajectory	Intercept	97.67417	15.03576	6.496	0.0000
	age	-12.48068	3.48212	-3.584	0.0003
	age2	0.81159	0.24857	3.265	0.0011
	age3	-0.01558	0.00575	-2.711	0.0067
	height	0.54634	0.06453	8.467	0.0000

The level (intercept) and change (age-linear, age^2-quadratic and age^3-cubic terms) of BP, standard errors and level of significance of the three height-adjusted BP trajectories.

2.8 **Table S8** Associations of baPWV in adulthood with BP trajectories from childhood to youth by sex

Trajectories	Male				Female			
	Unadjusted model		adjusted model		Unadjusted model		adjusted model	
	β(95%CI)	P value	β(95%CI)	P value	β(95%CI)	P value	β(95%CI)	P value
SBP trajectory groups								
Low-level	Reference		Reference		Reference		Reference	
Medium-level	53.0(39.2-66.8)	<0.001	45.9(29.8-62.0)	<0.001	74.1(60.0-8.2)	< 0.001	62.7(45.8-79.6)	< 0.001
High-level	128.6(103.9- 153.4)	<0.001	116.2(86.3-146.1)	<0.001	149.8(126.3-173.5)	< 0.001	114.6(84.9-144.4)	< 0.001
DBP trajectory groups								
Low-level	Reference		Reference		Reference		Reference	
Medium-level	56.2(42.8-69.6)	< 0.001	55.8(40.2-71.4)	< 0.001	54.5(37.9-71.0)	< 0.001	31.5(12.7-50.2)	0.001
High-level	217.6(181.3- 253.9)	< 0.001	220.8(181.3-260.3)	< 0.001	120.9(99.0-142.7)	< 0.001	91.3(63.4-119.2)	< 0.001
MAP trajectory groups								
Low-level	Reference		Reference		Reference		Reference	
Medium-level	49.9(36.7-63.0)	< 0.001	48.7(33.0-64.4)	< 0.001	54.9(40.4-69.4)	< 0.001	35.5(18.4-52.7)	< 0.001
High-level	249.3(213.3-285.3)	< 0.001	231.3(191.7-270.8)	< 0.001	131.3(108.8-153.8)	< 0.001	100.7(72.1-129.3)	< 0.001
PP trajectory groups								
Low-level	Reference		Reference		Reference		Reference	
Medium-level	27.8(14.2-41.4)	< 0.001	6.0(-8.6-20.6)	0.419	35.5(17.8-53.2)	< 0.001	11.1(-8.7-30.8)	0.273
High-level	62.0(21.2-91.7)	< 0.001	9.0(-23.6-41.6)	0.589	34.7(-8.8-78.1)	0.118	-24.6(-69.3-20.2)	0.283

Abbreviation: baPWV, brachial–ankle pulse wave velocity; SBP, systolic blood pressure; DBP, diastolic blood pressure. CI, confidence interval.

Dependent variables: baPWV. Adjusted Model: adjustments as in model2 plus time-dependent variables (body mass index, SBP, DBP, MAP and heart rate), childhood factors including father's education level and mother's education level (College or university=0, High school=1, Middle school=2 and Primary or less=3) and adulthood factors including family history of hypertension(Yes=1, No=0) , smokers(Yes=1, No=0), TC, TG, LDL-C, HDL-C, uric acid, and fasting blood glucose.
